# Vasculometabolic and Inflammatory Effects of Aldosterone in Obesity

**DOI:** 10.1210/clinem/dgaa356

**Published:** 2020-06-12

**Authors:** Charlotte D C C van der Heijden, Rob ter Horst, Inge C L van den Munckhof, Kiki Schraa, Jacqueline de Graaf, Leo A B Joosten, A H Jan Danser, Mihai G Netea, Jaap Deinum, Joost Rutten, Niels P Riksen

**Affiliations:** 1 Department of Internal Medicine, Radboud University Medical Center, Nijmegen, GA, the Netherlands; 2 Radboud Institute of Molecular Life Sciences (RIMLS), Radboud University Medical Center, Nijmegen, GA, the Netherlands; 3 Department of Medical Genetics, Iuliu Hațieganu University of Medicine and Pharmacy, Cluj-Napoca Romania; 4 Department of Internal Medicine, Erasmus Medical Center, Rotterdam, GD, the Netherlands; 5 Department for Genomics & Immunoregulation, Life and Medical Sciences 12 Institute (LIMES), University of Bonn, Bonn, Germany

**Keywords:** aldosterone, obesity, renin-angiotensin-aldosterone system, atherosclerosis, inflammation, metabolomics

## Abstract

**Context:**

Not all obese individuals develop cardiovascular disease (CVD). Hyperaldosteronism is suggested to cause inflammation and metabolic dysregulation, and might contribute to CVD development in obese individuals.

**Objective:**

We aimed to investigate the association of aldosterone concentrations with inflammation, metabolic disturbances, and atherosclerosis in overweight and obese individuals. Additionally, we measured renin concentrations to investigate whether the observed effects reflected general activation of the renin-angiotensin-aldosterone system (RAAS).

**Design:**

A cross-sectional cohort study (300-OB study) was conducted. Various inflammatory parameters, traits of the metabolic syndrome, lipidome and metabolome parameters, fat distribution, and carotid atherosclerosis were associated with plasma aldosterone and renin levels.

**Setting:**

The setting of this study was the Radboudumc (i.o. Radboudumc), the Netherlands.

**Patients:**

A total of 302 individuals with a body mass index greater than or equal to 27 kg/m^2^ participated.

**Main Outcome Measures and Results:**

Aldosterone was associated with various markers of inflammation and metabolic dysregulation, which partly differed from the associations observed for renin. Although both were associated with inflammatory cell numbers, only renin was associated with classical markers of systemic inflammation. Both were associated with the metabolic syndrome and hepatic steatosis. Of the traits that constitute metabolic syndrome, aldosterone, but not renin, was associated with triglyceride concentrations. Accordingly, aldosterone was associated with large very low-density lipoprotein particles; metabolomics studies further associated aldosterone with urate concentrations and derivatives of the linoleic acid metabolism pathway. Neither aldosterone nor renin was associated with atherosclerotic plaque thickness.

**Conclusions:**

Aldosterone is not an important driver of systemic inflammation in the obese, whereas aldosterone concentrations and metabolic dysregulation are strongly intertwined in these individuals. Although prospective studies are necessary to validate these results, the independent effects of aldosterone on carotid atherosclerosis appear modest.

Cardiovascular disease (CVD) is the number one cause of morbidity and mortality worldwide. One of its risk factors is obesity, a growing problem with epidemic proportions in Western as well as non-Western countries (1). Interestingly, not all obese individuals develop metabolic or cardiovascular complications, with approximately 20% to 30% of the obese population being “metabolically healthy” (2). This illustrates the complexity of the pathophysiological mechanisms linking obesity to CVDs. Among other factors, obesity-associated activation of the renin-angiotensin-aldosterone system (RAAS), in particular hyperaldosteronism, might contribute to CVD in obese individuals.

The development of obesity and hyperaldosteronism are closely connected. Hyperinsulinemia and high circulating levels of adipocytokines activate the sympathetic nervous system, which in turn activates the RAAS. The proinflammatory adipocytokine leptin further contributes to hyperaldosteronism by directly stimulating aldosterone production by the adrenals (3). Last, the adipose tissue itself produces aldosterone (4). Aldosterone in turn promotes the maturation and dysfunctional differentiation of adipocytes via the mineralocorticoid receptor, leading to local expansion of fat mass, enhanced production of adipocytokines, and insulin resistance (5), resulting in a maladaptive vicious cycle.

In the general population, aldosterone concentrations are associated with type 2 diabetes, statin use, and metabolic syndrome (6), and they predict future cardiovascular events (7). Preclinical models investigating the mechanisms through which aldosterone contributes to cardiometabolic dysfunction show that aldosterone is causally linked to insulin resistance (8), cardiac and vascular fibrosis (9), as well as inflammation and atherosclerosis (10). Importantly, aldosterone is not the only RAAS component that exerts biological actions with disadvantageous immunological and vasculometabolic consequences. Renin as well as angiotensin II have both been reported to promote inflammation and insulin resistance (11). Therefore, different components of the RAAS system could have additive effects on CVD risk in obesity, and the extent of RAAS activation might in part explain the variation in the individual risk of obese individuals to develop metabolic syndrome and CVD.

The 300-Obese (300-OB) cohort, one of the studies of the Human Functional Genomics Project, comprises a group of more than 300 individuals of Western-European ancestry with a body mass index (BMI) greater than or equal to greater than or equal to 27 kg/m^2^, aged between 55 and 80 years (12). The study was designed to discover novel pathways contributing to CVD in overweight and obese individuals. Using the comprehensive data set of the 300-OB study, we here provide a detailed assessment of the association of circulating aldosterone and renin levels with inflammation, metabolic dysregulation, and atherosclerosis in the obese to address the hypothesis that RAAS activation—in particular aldosterone—contributes to metabolic dysregulation and atherosclerosis development in these patients and unravel the underlying mechanisms.

## Methods

Extended methods are available in the Supplementary materials (13).

### The 300-Obese cohort

A total of 302 individuals aged 55 to 80 years were enrolled in the 300-OB study at the Radboud University Medical Center, Nijmegen, the Netherlands, between 2014 and 2016. Inclusion criteria consisted of age older than 55 years and a BMI greater than or equal to 27 kg/m^2^. Exclusion criteria consisted of a recent cardiovascular event (myocardial infarction, transient ischemic attack, and stroke < 6 months), a history of bariatric surgery or bowel resection, inflammatory bowel disease, renal dysfunction, increased bleeding tendency, use of oral or subcutaneous anticoagulant therapy, use of thrombocyte aggregation inhibitors other than acetylsalicylic acid and carbasalate calcium, or a contraindication for magnetic resonance imaging (MRI). Participants who used lipid-lowering therapy temporarily discontinued this medication 4 weeks before the measurements. Each individual provided written informed consent prior to participation in this study. The study was conducted according to the principles of the International Conference on Harmonization–Good Clinical Practice guidelines.

### Sample collection

Venous blood was drawn in the morning after an overnight fast.

### Self-reported sodium intake

Each individual kept a detailed food diary for 4 days prior to sampling. Participants filled out an online diary on the Dutch Nutrition Centre website (14) or a standardized paper diary that was subsequently entered into the online diary from the Dutch Nutrition Centre by the researcher. Afterward the nutritional content, including sodium intake, could be extracted for each day.

### Aldosterone and renin concentrations

Aldosterone and renin concentrations were measured in EDTA plasma and serum, respectively, by radioimmunometric procedures (Aldosterone RIA, IT1664, Demeditec Diagnostics GmbH; renin, Renin III generation, Cisbio Bioassay).

### Vascular measurements

We performed carotid ultrasound after an overnight fast or in the afternoon 6 hours after a standardized breakfast, after abstention from caffeine and smoking. The presence of carotid plaque was defined as focal thickening of the wall of at least 1.5× the mean carotid intima-medial thickness (cIMT) or a cIMT greater than 1.5 mm (15).

### Circulating mediators

Cytokines and circulating mediators were measured in EDTA plasma using enzyme-linked immunosorbent assay following the manufacturer’s instructions (R&D Systems). Interleukin (IL)-6 and IL-18 were measured by Simple Plex cartridges using the ELLA technology (Protein Simple).

### Lipidomics and metabolomics

Lipidomics were performed using a high-throughput nuclear magnetic resonance metabolomics platform (Nightingale’s Biomarker Analysis Platform) (16). General Metabolomics performed flow-injection electrospray time-of-flight mass spectrometry to identify metabolic features based on mass to charge ratio (m/z). In total, 1339 m/z signals were assigned to one or more metabolites.

### Assessment of fat distribution and hepatic steatosis

Abdominal fat distribution and liver fat content were determined by MRI and proton magnetic resonance spectroscopy (MRS), respectively.

### Statistics

Aldosterone and renin as well as outcome parameters were normalized using rank based inverse normal transformation (INT). Aldosterone and renin levels were compared to sets of measurements (eg, metabolomics, circulating cytokine levels). All comparisons were corrected for multiple testing (Benjamini-Hochberg procedure) within each set of measurements; a false discovery rate (FDR) of less than 0.05 was considered significant, and findings with an FDR of less than 0.1 are described. β-Coefficients represent the correlation of the normalized data. The covariates included in the analysis are age, sex, BMI, smoking, systolic and diastolic blood pressure, and season, and are further described in the Supplementary materials (13). Data are plotted as scatterplots with Loess curves. Additional analyses (Tables 1 and 2) were performed on participants not taking antihypertensives (55% of the cohort), dividing them into tertiles based on aldosterone (or renin) levels. Normalized outcome parameters were compared between the lowest and highest tertiles with analysis of covariance correcting for the covariates described above. Benjamini-Hochberg multiple test–corrected *P* values are reported.

**Table 1.** Subgroup analysis in those without antihypertensives, comparing lowest to highest aldosterone tertiles

Outcome parameter	Lowest tertile (mean [SD])	Highest tertile (mean [SD])	*P*
Peripheral blood cell composition			
Leukocytes, 10^9^/L	5.38 (1.16)	6.39 (1.72)	.001
Neutrophils, 10^9^/L	3.06 (0.99)	3.53 (1.27)	.032
Lymphocytes, 10^9^/L	1.65 (0.06)	2.11 (0.07)	< .001
Monocytes, 10^9^/L	0.45 (0.12)	0.52 (0.18)	.062
Circulating markers			
VEGF, pg/mL	35.93 (20.41)	62.34 (74.53)	.028
Metabolic syndrome score	1.96 (0.94)	2.53 (1.17)	.002
Metabolic markers			
Urate, mmol/L	0.35 (0.08)	0.38 (0.09)	.040
Triglycerides, mmol/L	1.47 (0.58)	1.74 (0.67)	.034
XL VLDL, mmol/L	0.069 (0.066)	0.091 (0.071)	.028
Metabolomics			
Lineoleic acid	6.97 (0.10)	7.03 (0.12)	.010
Arachidonic acid	5.93 (0.11)	6.01 (0.13)	.010
Adrenic acid	4.93 (0.14)	5.03 (0.16)	.010
Docosapentaenoic acid	5.28 (0.15)	5.38 (0.20)	.010
Prostaglandin F2A	4.36 (0.06)	4.39 (0.07)	.015
Leukotriene B4	4.43 (0.07)	4.47 (0.07)	.015
Liver fat	0.073 (0.096)	0.107 (0.128)	.13
Indicators of atherosclerosis			
Plaque presence, %	44	49	.57
Plaque thickness, cm	2.25 (1.04)	2.11 (0.48)	.79
cIMT, µm	769.5 (133.5)	788.2 (148.7)	.79
PWV, m/s	9.28 (1.90)	8.95 (1.71)	.79

Abbreviations: cIMT, carotid intima-medial thickness; PWV, pulse wave velocity; XL VLDL, extra-large very low-density lipoprotein.

**Table 2.** Subgroup analysis in those without antihypertensives, comparing lowest to highest renin tertiles

Outcome parameter	Lowest tertile (mean [SD])	Highest tertile (mean [SD])	*P*
Peripheral blood cell composition			
Leukocytes	5.68 (1.40)	6.31 (1.57)	.09
Neutrophils	3.12 (0.96)	3.68 (1.17)	.01
Lymphocytes	1.85 (0.55)	1.89 (0.57)	.93
Monocytes	0.48 (0.14)	0.51 (0.15)	.27
Platelets	229.5 (51.1)	238.8 (53.7)	.23
Circulating markers			
IL-6, pg/mL^*a*^	2.13 (1.55-2.87)	2.45 (1.70-3.42)	.17
IL18BP, ng/mL	16.77 (5.15)	17.23 (4.14)	.12
Adiponectin, µg/mL	5.20 (2.41)	4.67 (2.38)	.17
Metabolic syndrome score	2.02 (1.06)	2.24 (1.19)	.12
Metabolic markers			
Urate	0.36 (0.08)	0.35 (0.09)	.37
Triglycerides, mmol/L	1.53 (0.62)	1.61 (0.68)	.36
Glucose, mmol/L	5.25 (0.60)	5.76 (1.75)	.03
Fat distribution			
Liver fat	0.079 (0.113)	0.093 (0.087)	.57
VAT	92.59 (25.43)	110.25 (34.56)	.02
Indicators of atherosclerosis			
Plaque presence, %	47	53	.57
Plaque thickness, cm	2.32 (1.01)	2.11 (0.48)	.47
cIMT, µm	794.8 (175.1)	775.9 (125.9)	.47
PWV, m/s	9.43 (1.53)	9.25 (1.92)	.47

Abbreviations: cIMT, carotid intima-medial thickness; IL-6, interleukin 6; IL18BP, interleukin-18-binding protein; VAT, visceral adipose tissue.

^a^Median (25th-27th percentile).

## Results

### Baseline parameters

**Figure 1.**
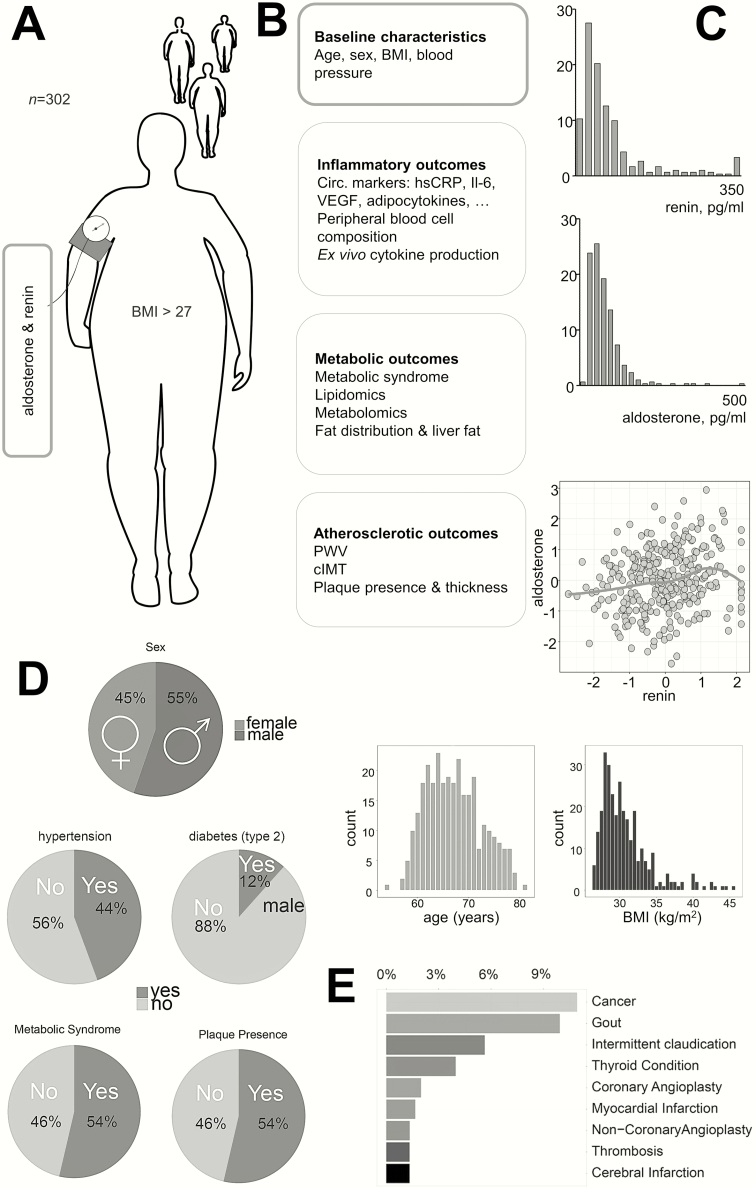
Overview of the 300-Obese cohort. A, We measured circulating aldosterone and renin in 302 individuals with a body mass index of 27 or greater. B, All individuals were extensively profiled. C, Histogram showing aldosterone and renin levels. D, Graphical representation of baseline characteristics of the cohort. E, Bar chart showing the most prevalent comorbidities in the cohort.

The study measurements and baseline characteristics of the study participants are described in Fig. 1A and 1B, respectively. Aldosterone concentrations (median 50.0 pg/mL, interquartile range 31.0-78.9 pg/mL) were comparable to previously reported values (6, 17). Renin concentrations showed a similar distribution (median 39.2 pg/mL, interquartile range 17.3-76.3 pg/mL), and were significantly correlated to aldosterone levels (r = 0.20, *P* < 1*10^–3^) (Fig. 1C). Data on comorbidity are presented in Fig. 1D. Forty-five percent of the population used antihypertensive medication: calcium antagonists (10%), β-blockers (22%), diuretic antihypertensives (23%), or renin-angiotensin system inhibitors (29%) (mineralocorticoid receptor antagonists were used by 1% of the population). Self-reported sodium intake showed a (weak) association with aldosterone levels (σ = –0.103, *P* = .08), but not with renin levels (σ = –0.002, *P* = .97).

### Renin, but not aldosterone, associates with classical circulating markers of inflammation

**Figure 2.**
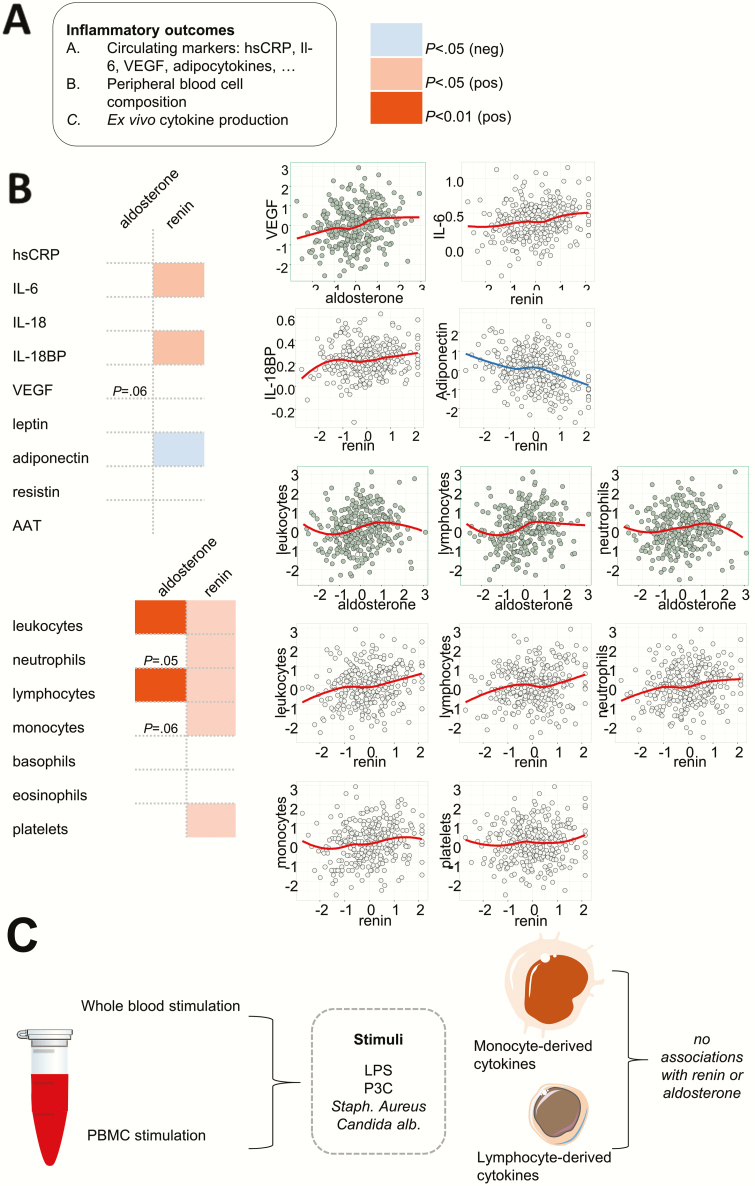
Associations of aldosterone and renin with markers of inflammation. A, Aldosterone and renin associated with different circulating markers of inflammation. B, The association of aldosterone and renin with inflammatory cell subtypes largely overlapped. C, Whole blood and peripheral blood mononuclear cells were isolated from all individuals and stimulated ex vivo with various stimuli, but no association of ex vivo cytokine production and aldosterone concentrations (or renin) was observed.

Aldosterone did not correlate significantly with any of the circulating markers. However, it displayed a trend to correlate with vascular endothelial growth factor A (VEGF-A) concentrations (β = .14, *P* = .06). Renin was significantly associated with the classical inflammatory markers interleukin (IL)-6 (β = .16) and IL-18 binding protein (IL18BP) (β = .17) and negatively with adiponectin (β = –.16). Neither aldosterone nor renin were associated with leptin or resistin levels. This is depicted in Fig. 2A.

### Aldosterone and renin associate with leukocyte counts

Aldosterone and renin were both associated with total leukocyte (β = .18 and β = .17, respectively), and lymphocyte counts (β = .18 and β = .14, respectively). Renin also was significantly associated with neutrophil and monocyte counts (β = .13 and β = .15); aldosterone showed a strong trend to correlate with these cell types (β = .13 and β = .11, respectively). Renin was also associated with thrombocyte counts (β = .19) (Fig. 2B).

### Neither aldosterone nor renin associate with ex vivo cytokine or lactate production

Whole blood and peripheral blood mononuclear cells from all individuals were stimulated ex vivo with various stimuli (Fig. 2C). Neither aldosterone nor renin were associated with cytokine production. Accordingly, lactate production on ex vivo stimulation, an indicator of glycolytic activity of immune cells, was not associated with aldosterone or renin concentrations (data not shown).

### Aldosterone and renin both are associated with markers of metabolic dysregulation

**Figure 3.**
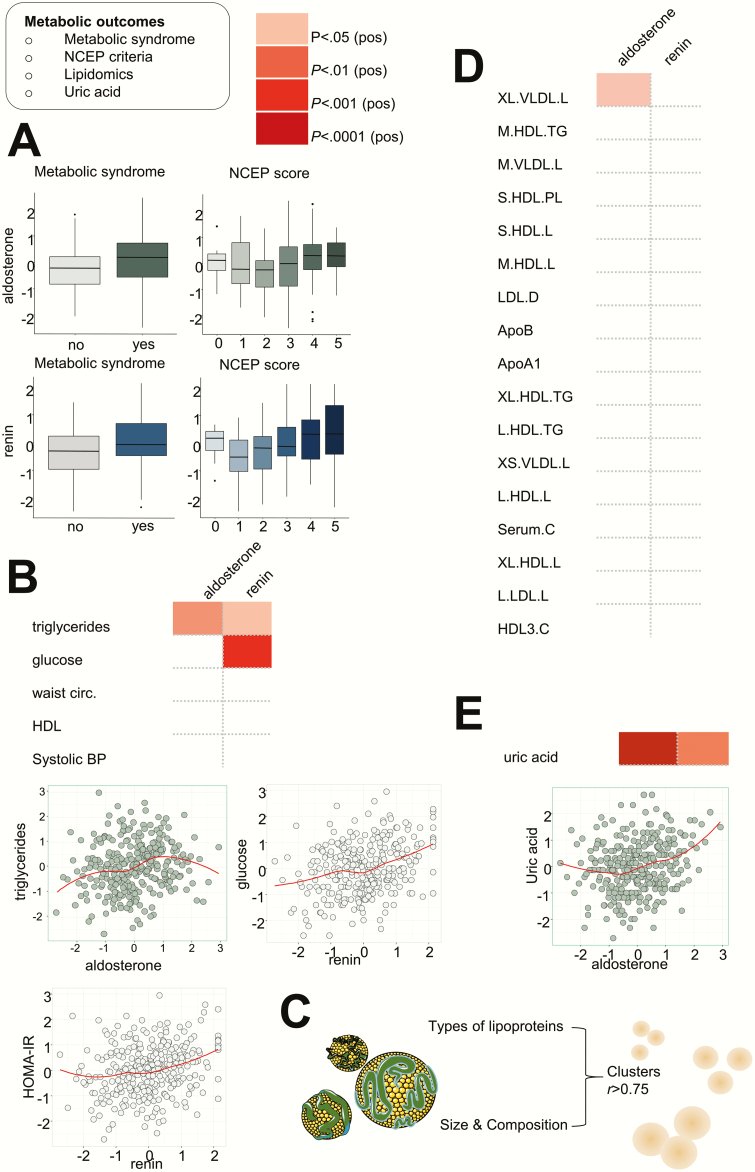
Associations of aldosterone and renin with metabolic syndrome and metabolic derangements. A, Aldosterone was associated with the presence of metabolic syndrome, in line, patients with the metabolic syndrome had higher circulating aldosterone levels. Similar associations were observed for renin. Both aldosterone and renin associated with the number of positive NCEP criteria for metabolic syndrome. B, Aldosterone was associated with triglyceride levels, whereas renin associated with glucose levels. In line, renin associated with the HOMA-IR. C, Lipidomics was performed, and lipid particles clustered. D, Aldosterone was associated with the cluster of large to extra-large VLDL particles. E, Last, we observed a striking association of aldosterone with uric acid. HOMA-IR, Homeostatic Model Assessment for Insulin Resistance; NCEP, National Cholesterol Education Program, VLDL, very low-density lipoprotein.

Aldosterone and renin are both strongly associated with the presence of metabolic syndrome, and patients with metabolic syndrome displayed higher aldosterone and renin concentrations. In line with this, aldosterone and renin associated with metabolic syndrome scores based on the National Cholesterol Education Program (NCEP) criteria (Fig. 3A). Interestingly, the association with metabolic syndrome was established through different traits for aldosterone and renin. Aldosterone was associated with triglyceride levels (β = .16), whereas renin most strongly associated with glucose levels (β = .24) (Fig. 3B). Renin, but not aldosterone, was associated with the Homeostatic Model Assessment for Insulin Resistance (β = .15), indicating insulin resistance (Fig. 3B). Extensive lipidomic analysis provided information on lipid particle size and composition (Fig. 3C). Because of the strong correlations between lipid particles, they were grouped into associated clusters (clusters include particles with an r > 0.75). Using selected representative particles per cluster, we found that aldosterone was associated with large and extra-large very low-density lipoprotein (VLDL) particles (β = .17), whereas renin was not associated with lipid clusters (Fig. 3D). Last, aldosterone showed a significant association with urate concentrations (β = .24) (Fig. 3E); the association of renin with this metabolite was less strong (β = .19).

### Both aldosterone and renin associate with liver fat on magnetic resonance spectroscopy

**Figure 4.**
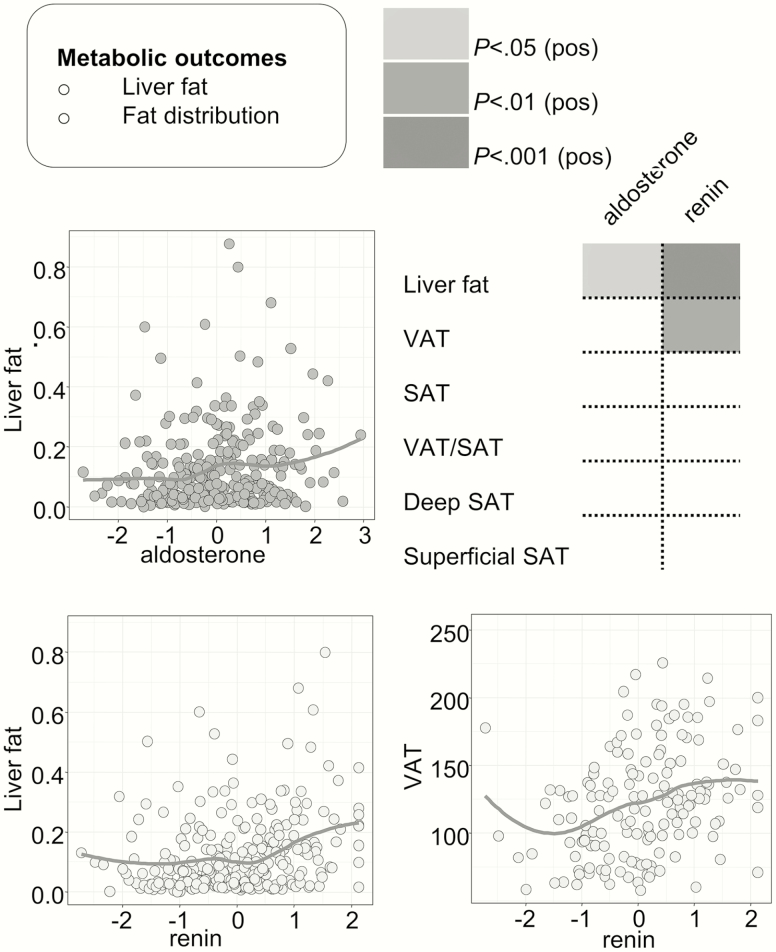
Associations of aldosterone and renin with abdominal fat distribution and liver fat. Aldosterone, and more strongly renin, both were associated with the amount of liver fat on magnetic resonance spectroscopy. Renin was also associated with the amount of visceral adipose tissue on magnetic resonance imaging.

Aldosterone, and to a greater extent renin, was associated with the amount of liver fat on MRS. Renin concentrations also associated with the total amount of visceral adipose tissue on MRI (Fig. 4).

Metabolome profiling shows a strong association of aldosterone with various fatty acids in the α-linolenic and linoleic acid metabolism pathway.

**Figure 5.**
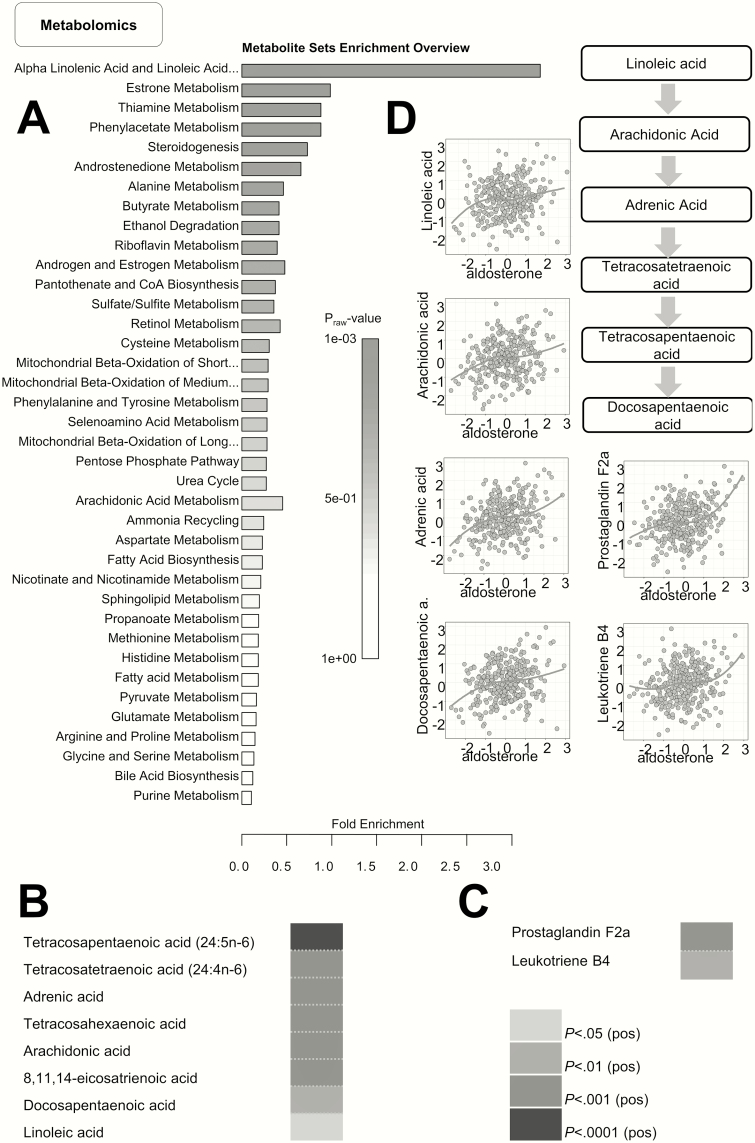
Metabolomics reveal that aldosterone levels associate with various metabolites in the linoleic acid metabolism pathway. A. Metabolite set enrichment analysis of all metabolites showing a significant positive association revealed linoleic acid metabolism as one of the most enriched pathways. B, Correlation of aldosterone with various intermediates of the linoleic acid metabolism, that is, C, arachidonic acid and its derivatives. D, Graphical representation of the linoleic acid pathway showing scatterplots of correlation of aldosterone with its derivatives.

Metabolome assessment revealed that out of 1394 plasma metabolites, 165 were positively associated with aldosterone after multiple test correction, but none with renin. A list of these 165 metabolites is presented in Supplementary Table 2 (13). As expected, various steroids and steroid precursors were positively associated with aldosterone levels, among which were androstenedione, androsterone glucuronide, and pregnenolone. Furthermore, we identified several fatty acids of medium to very long chain length and saturated as well as unsaturated nature, in addition to phospholipids and eicosanoids. Subsequently, we performed a pathway enrichment analysis of the 165 metabolites. Although no pathways were significantly enriched, there was a trend toward enrichment of α-linolenic and linoleic acid metabolism pathway (raw *P* value = .001, FDR = .118), shown in Fig 5A. Metabolites contributing to enrichment of this pathway are depicted in Fig 5B, and mainly clustered in the linoleic acid, rather than the α-linolenic acid metabolism pathway (Fig. 5C)

### Renin and aldosterone do not associate with carotid atherosclerosis

Neither aldosterone nor renin associated with pulse wave velocity or cIMT. Aldosterone and renin concentrations did not predict the presence of an atherosclerotic plaque. In patients with atherosclerotic plaques, neither aldosterone nor renin was associated with plaque thickness (data not shown).

### Confirmation of findings in individuals without antihypertensives

Forty-five percent of the study population used antihypertensives. Of those, patients using diuretics displayed significantly higher aldosterone concentrations than those not using these drugs (aldosterone 78 ± 63 pg/mL vs 48 ± 57 pg/mL). Therefore, we validated all significant and trending associations (*P* < .1) described in previous paragraphs in those without antihypertensive drugs (n = 166), dividing this subgroup into tertiles based on plasma aldosterone levels (Table 1) and plasma renin levels (Table 2) and comparing outcomes in lowest to highest aldosterone/renin tertiles. Self-reported sodium intake did not differ significantly between the lowest and highest aldosterone tertiles (2.5 ± 1.2 vs 2.2 ± 1.1 g/24 hours, *P* = .15), or renin tertiles (2.5 ± 1.2 vs 2.4 ± 1.2 g/24 hours, *P* = .81). Comparable to the association in the complete study cohort, we observed a significant, albeit weak, association between renin and aldosterone levels (Spearman rho 0.194, *P* = .012) in this subgroup. We confirm associations of aldosterone with white blood cell counts, VEGF, metabolic syndrome scores, triglycerides, and extra-large VLDL, urate, and linoleic acid and its derivatives (Table 1). Moreover, similar to our observations in the complete cohort, we did not observe an association with classical markers of inflammation (not shown) or indicators of atherosclerosis (Table 1). Last, we provide coefficients and *P* values for the linear associations between aldosterone/renin and outcome parameters in those without antihypertensives and specifically diuretic antihypertensives in Supplementary Table 1 (13).

## Discussion

This study provides a comprehensive assessment of the association of aldosterone concentration with inflammation, metabolic dysregulation, and atherosclerosis in a well-characterized cohort of more than 300 overweight and obese individuals. Based on previous preclinical research (10), we hypothesized that aldosterone—partly independent from renin—induces systemic inflammation and activation of circulating immune cells and thereby contributes to the development of vasculometabolic dysregulation in the obese. Although aldosterone showed a strong association with leukocyte numbers, it was not associated with systemic inflammatory markers, nor with cytokine production capacity of circulating immune cells. Therefore, we conclude that in obese individuals, aldosterone is not a major driver of systemic inflammation and immune system activation. We did however reveal specific associations of aldosterone, and not renin, with VLDL particles, linoleic acid metabolism, and urate, which provide novel clues about the pathogenic effects of aldosterone in obesity.

Obesity is characterized by low-grade inflammation with a multifaceted pathophysiology (18). Landmark trials have provided irrefutable evidence that inflammation is causally related to atherosclerotic CVD (19), underscoring the importance of the identification of common pathways driving inflammation in those at high CVD risk. Although murine models of hyperaldosteronism suggest proinflammatory effects of aldosterone (10, 20), and we previously showed long-term activation of human monocyte–derived macrophages after aldosterone exposure (21), we did not find evidence of proinflammatory effects of aldosterone in this cohort of obese individuals. These findings are in line with our recent findings that there are no significant differences in circulating IL-6 and high-sensitivity C-reactive protein levels or ex vivo cytokine production in a cohort of patients with primary aldosteronism compared to matched hypertensive controls (22). Moreover, no association of aldosterone with high-sensitivity C-reactive protein was found in a large population-based study (17). There are several potential explanations for these discrepant results. First, in preclinical hyperaldosteronism models the aldosterone concentration is often higher that the in vivo concentration in humans. Second, many of the conclusions on the effects of mineralocorticoid excess are extrapolated from MR knockout models. It is to be debated whether the magnitude of the biological effect of the absence of mineralocorticoid signaling would compare to—relatively subtle—effects of MR overstimulation. Third, many of these models are confounded by the effects of mineralocorticoids on blood pressure, which in itself affects cardiovascular health. Last, our present study is unique in that it explores the effect of aldosterone on inflammation in the context of obesity, which in itself promotes inflammation. Our findings provide evidence that in this context, aldosterone does not impose major additional proinflammatory effects.

Surprisingly, we could not confirm the previously reported association of aldosterone with the proinflammatory adipocytokine leptin (3), which is probably because in our cohort of obese individuals the variation in BMI and therefore leptin levels was small. Renin, however, was associated with the proinflammatory cytokine IL-6, a cytokine causally related to atherogenesis and CVD (23-25), and showed a negative association with the anti-inflammatory adiponectin, which has been described before (26). Of interest, we observed a trending association between aldosterone concentrations and VEGF-A, a pivotal regulator of angiogenesis (27). Although its role in CVD is inconclusive, VEGF-A has been suggested to increase monocyte chemotaxis and atherosclerotic plaque neovascularization (28). Recently, VEGF-A was shown to directly induce aldosterone synthase activity in adrenal cells (29). Alternatively, aldosterone could affect VEGF-A production because aldosterone induced VEGF-A expression in neutrophils (30) and the expression of VEGF family members in vascular smooth muscle cells (31).

Next, we investigated the association of aldosterone with metabolic derangements. We confirmed its association with metabolic syndrome (6). Surprisingly, aldosterone and renin were associated with metabolic syndrome via different traits. Aldosterone was associated with triglyceride levels, in line with previous reports (6). Renin concentrations, on the other hand, were associated most strongly with glucose levels and insulin resistance, confirming previous reports (32). The observation that renin, but not aldosterone, negatively associates with adiponectin—a potent insulin sensitizer (33), might explain part of this association. For aldosterone, a lipidomics approach pinpointed that large and extra-large VLDL particles most significantly contributed to the observed association with triglycerides. VLDL, a triglyceride-rich remnant particle, has been convincingly linked to CVD. In a large mendelian randomization trial, elevated nonfasting remnant cholesterol was associated with inflammation and ischemic heart disease (34). Moreover, VLDL-associated apolipoproteins were the strongest predictors of cardiovascular events in a prospective population-based study (35). Interestingly, an increasing amount of in vitro data suggest that VLDL can activate aldosterone production in the adrenals (36), suggesting that hyperaldosteronism results from, rather than causes, hypertriglyceridemia. This fits with our observation that renin is not associated with VLDL, excluding an effect of general RAAS system activation on VLDL concentrations. Further supporting this hypothesis is the clinical observation that statin use lowers aldosterone production independently of changes in renin or potassium (37).

Two other findings in this study are of particular interest for CVD. First, we observed an association of aldosterone with urate levels, a metabolite that is linked to inflammation and cardiovascular morbidity and mortality (38). Although data from mechanistic and population-based studies are conflicting (39, 40), ours suggest that in an obese population, aldosterone levels are associated with urate levels independently of classical CVD risk factors. Second, a metabolomics approach revealed that aldosterone was associated with fatty acids in the linoleic acid metabolism pathway. Linoleic acid, an ω-6 essential fatty acid, is the most abundant polyunsaturated fatty acid in humans. In CVD, its role appears mainly protective (41). Interestingly, oxidative derivatives of linoleic acid were previously shown to stimulate aldosterone production in vitro, and to be associated with aldosterone levels in a small cohort of healthy adults (42). Because linoleic acid is an essential amino acid, these findings also raise the question whether its dietary intake could directly affect aldosterone levels. Linoleic acid has several important downstream derivatives. One of the most well known is arachidonic acid, with which aldosterone showed a clear association (43). Although the direct inflammatory activity of arachidonic acid seems limited, it is the precursor of inflammatory eicosanoids. Among these is leukotriene B4, which we identified to be associated with aldosterone. Leukotriene B4, a potent chemoattractant mainly recruiting neutrophils, is associated with various inflammatory processes and mechanistic studies established its role in atherosclerosis (44, 45).

Last, we assessed the interaction of aldosterone and renin with carotid artery atherosclerosis. In animal models, RAAS activation, in particular induction of angiotensin II (46) and aldosterone (10), has been associated with accelerated atherogenesis. In patients with essential hypertension, aldosterone levels correlate to markers of preclinical atherosclerosis (40). Accordingly, we found that patients with primary hyperaldosteronism display enhanced arterial wall inflammation compared to hypertensive controls, suggestive of active (pre)atherosclerotic disease (22). In the present study, neither aldosterone nor renin was associated with markers of atherosclerosis in obese individuals. However, it is important to realize that our cohort differs significantly from the models and studies mentioned. Most important, the prevalence of risk factors for atherosclerosis, for example, insulin resistance and dyslipidemia, is significantly higher in obese individuals, which is likely to reduce the relative impact of renin and aldosterone on atherosclerosis development. Moreover, the design of our model and the age of our participants do not enable us to prospectively investigate the relation between RAAS activation and atherosclerosis development. Importantly, most participants in this cohort had no clinical symptoms of atherosclerotic CVD. Because symptomatic atherosclerosis is more strongly associated with inflammation than asymptomatic atherosclerosis, and inflammation is particularly important in destabilization of atherosclerotic plaques (47, 48), it is possible that RAAS activation induces inflammatory changes in atherosclerotic plaques, rather than the development of asymptomatic atherosclerosis per se.

Our study exemplifies the complex interaction of aldosterone, the RAAS, and obesity. Our findings result in 4 important hypotheses. First, because the studied associations differ between aldosterone and renin, our findings underscore the hypothesis that aldosterone in obese individuals is regulated by factors that are in part renin independent. Second, it has recently been hypothesized that aldosterone synthesis by the adrenals can be induced by inflammatory (ie, VEGF-A) and metabolic compounds (ie, VLDL), which increases the complexity of studying the independent effects of aldosterone on CVD in individuals with inflammatory and vasculometabolic derangements (such as obese individuals). However, numerous preclinical studies, with limited impact of confounding factors, described the immunologic and metabolic effects of aldosterone. Therefore, we thirdly hypothesize that part of the established associations are bidirectional, with aldosterone and common inflammometabolic indices intertwined in a vicious cycle. Fourth, based on our findings we hypothesize that different RAAS components could have different, additive effects on vasculometabolic health. Further elucidation of these mechanisms could contribute to the development of individualized pharmacological strategies for obese patients with increased cardiovascular risk.

Several limitations of our study require consideration. Almost half of our studied participants used antihypertensive medication, which affects the RAAS. Also, levels of RAAS hormones are dependent on sodium intake, diurnal rhythms, circulating volume, and exercise (49). As expected, self-reported sodium intake correlated negatively (albeit weakly) with aldosterone levels in our cohort. Although we aimed to reduce the influence of diurnal rhythms on hormone levels by obtaining all biomaterial in the morning, we cannot fully exclude variation due to the timing of sampling. However, because mineralocorticoid receptor antagonists were used in only 1% of our cohort, the height of circulating aldosterone levels reflects bioactivity at the MR, which is independent from the factors that determine these aldosterone levels. Importantly, we confirmed our findings in a subanalysis of the 55% of our cohort not using antihypertensives and comparable in salt intake, further underscoring the robustness of the associations of aldosterone and the reported outcome parameters. A second limitation of our study is the cross-sectional nature of our measurements, which does not enable us to determine the directionality of the observed effects. Mendelian randomization approaches would help to unravel whether RAAS hormones are causally related to the outcome parameters measured, whereas prospective studies would be the gold standard. Last, we did not measure angiotensin II levels in our cohort. Angiotensin II is a potent effector of renal and extrarenal RAAS effects, and therefore some associations—especially those of renin—might be exerted via angiotensin II.

In conclusion, our study revealed that aldosterone is associated with inflammatory cell counts and metabolic dysregulation in obese individuals, in part independently of renin. The increasing scale of the worldwide obesity problem warrants understanding of all the mechanisms that affect CVD risk in the obese. Importantly, because medication blocking the specific targets of aldosterone and other RAAS hormones are readily available, the potential therapeutic consequences of our findings are discernible. Although future studies are needed to investigate the directionality of the effects, our data reveal several novel pathways that could link aldosterone and RAAS activation to development of CVD in the obese.

## Abbreviations

300-OB: 300-Obese
BMI: body mass index
cIMT: carotid intima-medial thickness
CVD: cardiovascular disease
FDR: false discovery rate
MRS: magnetic resonance spectroscopy
NCEP: National Cholesterol Education Program
RAAS: renin-angiotensin-aldosterone system
VEGF: vascular endothelial growth factor
VLDL: very low-density lipoprotein

## Additional Information

***Disclosure Summary:*** The authors have nothing to disclose.

***Data Availability:*** The data sets generated during and/or analyzed during the current study are not publicly available but are available from the corresponding author on reasonable request.
